# 

**Published:** 1886-08

**Authors:** 


					﻿Entered at the Post Office, at Austin, Texas, as Second Class Mail Matter
Vol. II.
AUGUST. 1886.
NO. 2
DANIEL'S
MEDICAL
JOURNAL
F. E. DANIEL., M. D., Editor and Publisher, AUSTIN, TEXAS.
Northern office, 1217 Filbert Street, Philadelphia.
Steam Book and Jor Printer, Austin, Texas
				

